# Supplementary material for: Combined Dopamine and Grape Seed Extract-Loaded Solid Lipid Nanoparticles: Nasal Mucosa Permeation, and Uptake by Olfactory Ensheathing Cells and Neuronal SH-SY5Y Cells
Source: Pharmaceutics. 2023 Mar 8;15(3):881. doi: 10.3390/pharmaceutics15030881 (PMC10059967; doi:10.3390/pharmaceutics15030881)
Supplement: Supplementary file 1 [file pharmaceutics-15-00881-s001.zip › pharmaceutics-2187364-supplementary.pdf]

SUPPLEMENTARY MATERIALS

FOR

Combined Dopamine and Grape Seed Extract-Loaded Solid Lipid Nanoparticles: Nasal mucosa permeation, and Uptake by Olfactory Ensheathing Cells and neuronal SH-SY5Y Cells

Adriana Trapani[1,*], Stefano Castellani[2], Lorenzo Guerra[3], Elvira De Giglio[4], Giuseppe Fracchiolla[1], Filomena Corbo[1], Nicola Cioffi[4], Giuseppe Passantino[5], Maria Luana Poeta[3], Pasqualina Montemurro[2], Rosanna Mallamaci[3], Rosa Angela Cardone[3] and Massimo Conese[6]

[1] Department of Pharmacy-Drug Sciences, University of Bari "Aldo Moro", 70125-Bari, Italy
[2] Department of Precision and Regenerative Medicine and Ionian Area (DiMePRe-J)-"University of Bari "Aldo Moro", 70125-Bari, Italy
[3] Department of Biosciences, Biotechnologies and Environment, University of Bari "Aldo Moro", 70125-Bari, Italy
[4] Department of Chemistry, University of Bari "Aldo Moro", 70125-Bari, Italy
[5] Department of Veterinary Medicine, Pathological Anatomy, University of Bari "Aldo Moro", 70125-Bari, Italy
[6] Department of Clinical Experimental Medicine, University of Foggia, 71122 Foggia, Italy
* Correspondence: adriana.trapani@uniba.it; Tel.: +39-080-5442114

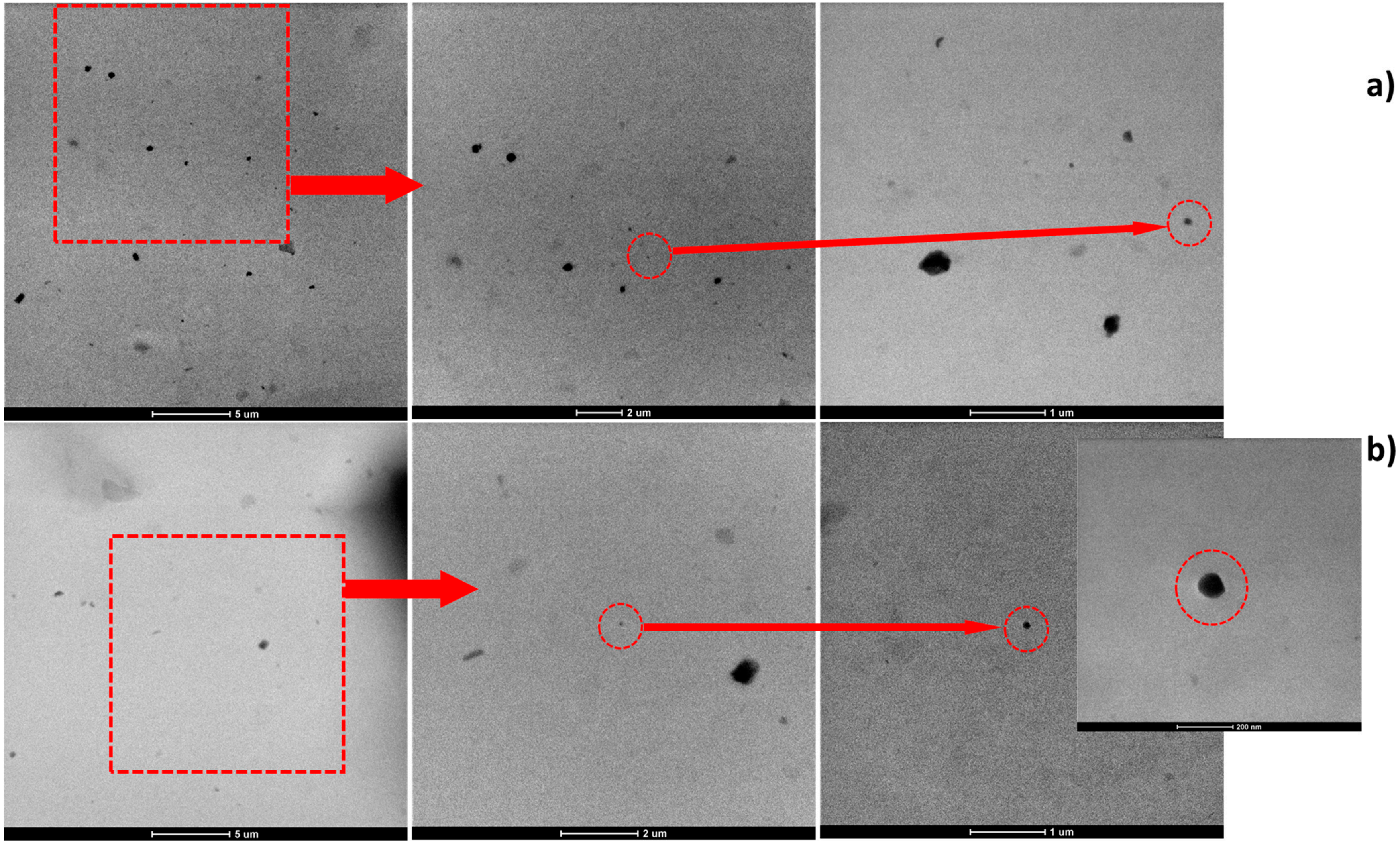

Figure S1. Sequential Transmission Electron Microscopy images of SLNs acquired at increasing magnifications, from left to right. In low-magnification images, the visualization of the smaller particles is poor. They become evident and quantifiable only at sufficiently high magnification, in small-sized analysis spots, where they are far enough from bigger particles (a). The size estimation of the present study was based on multiple images and several fields were acquired at the highest possible magnification, like in the inset of panel (b).

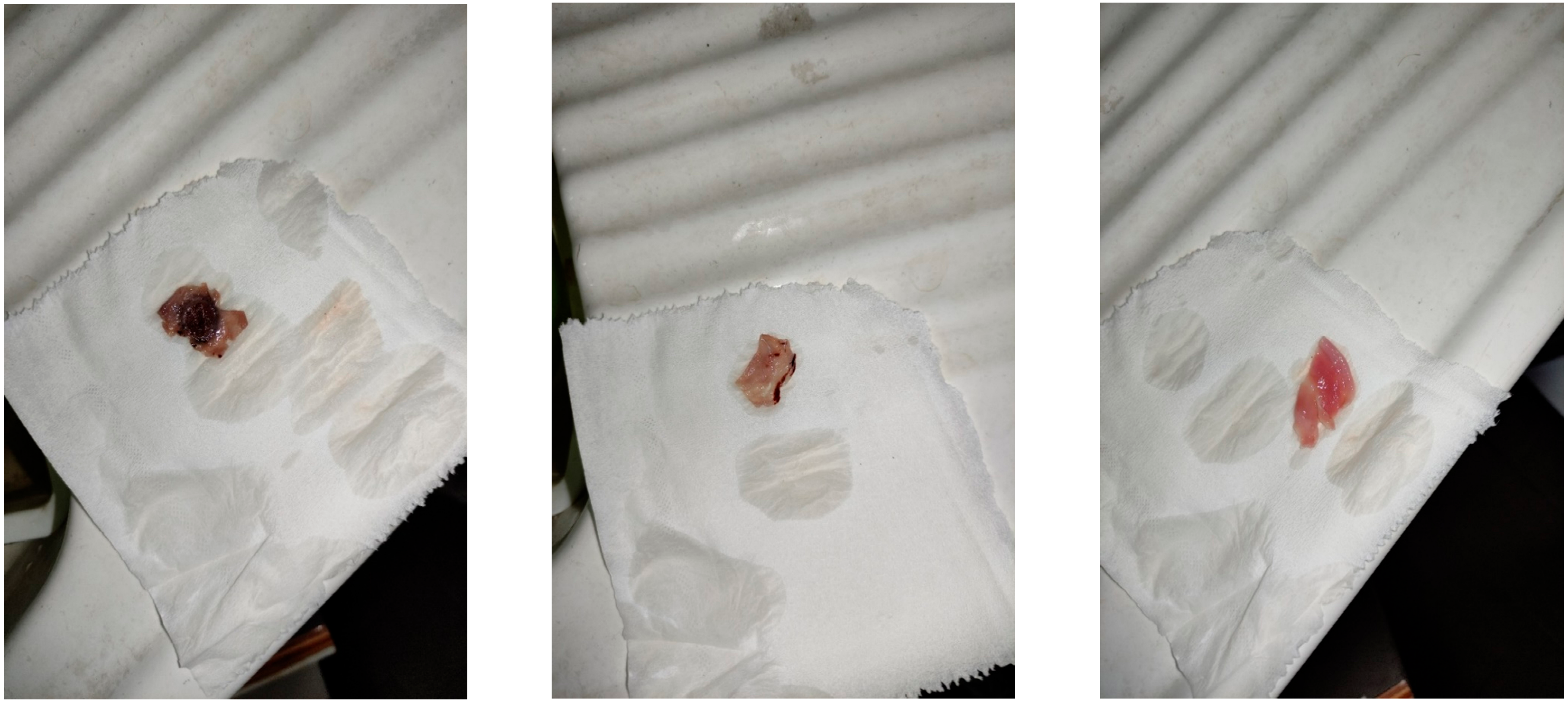

(**a**) (**b**) (**c**)

Figure S2. Nasal porcine mucosa visualization after experiment with DA-co-GSE-SLNs exposed to Franz cell (**a**), GSE-ads-DA-SLNs exposed to Franz cell (**b**) and control porcine mucosa without any treatment (**c**).
